# Supplementary material for: Proteomic and Biochemical Approaches Elucidate the Role of Millimeter-Wave Irradiation in Wheat Growth under Flooding Stress
Source: Int J Mol Sci. 2022 Sep 8;23(18):10360. doi: 10.3390/ijms231810360 (PMC9499361; doi:10.3390/ijms231810360)
Supplement: Supplementary file 1 [file ijms-23-10360-s001.zip › rev Supplemental Figures.pdf]

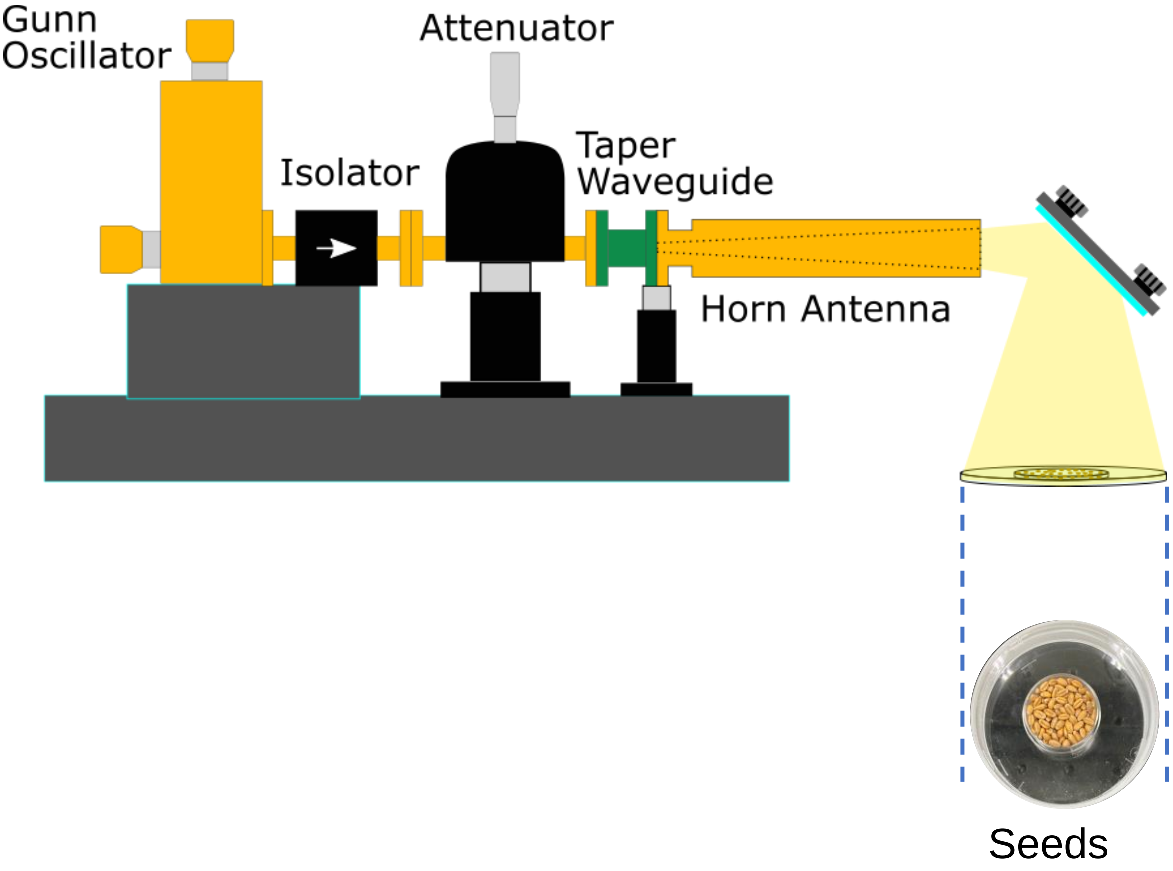

Figure S1: Device for millimeter-wave irradiation emission used in this study. Millimeter waves were generated from Gunn oscillator. Plate which installed with wheat seeds was vertically fixed at a distance of 15 cm from the center of oscillator.

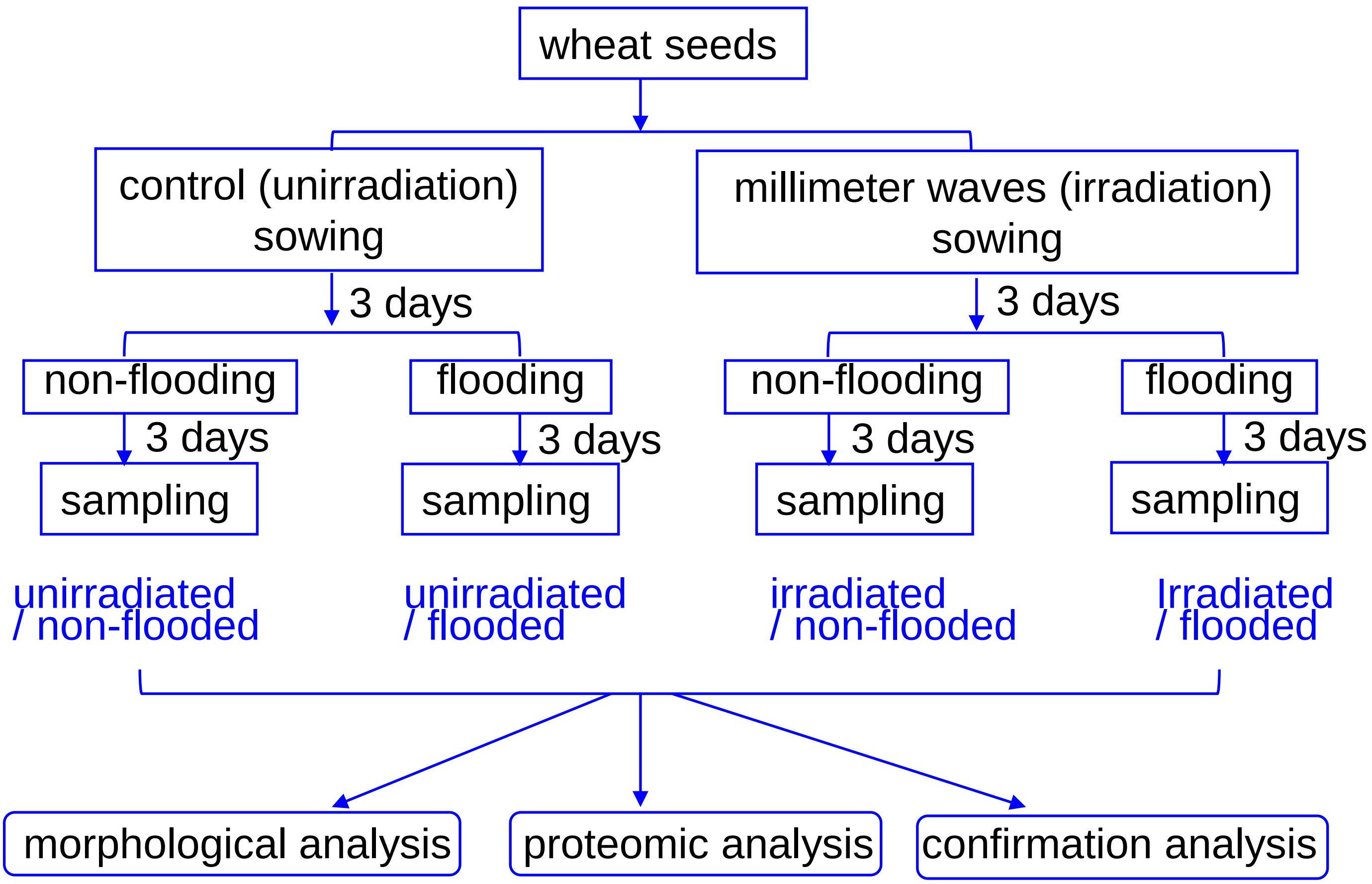

Figure S2: Experimental design to examine the effects of millimeter-wave irradiation on wheat under flooding stress. To investigate the potential effects of millimeter-wave irradiation on wheat, seeds were irradiated both without and with millimeter waves at various power levels and for various durations. Wheat seedlings were investigated with morphological and proteomic and confirmation analyses. All experiments were performed with 3 independent biological replicates.

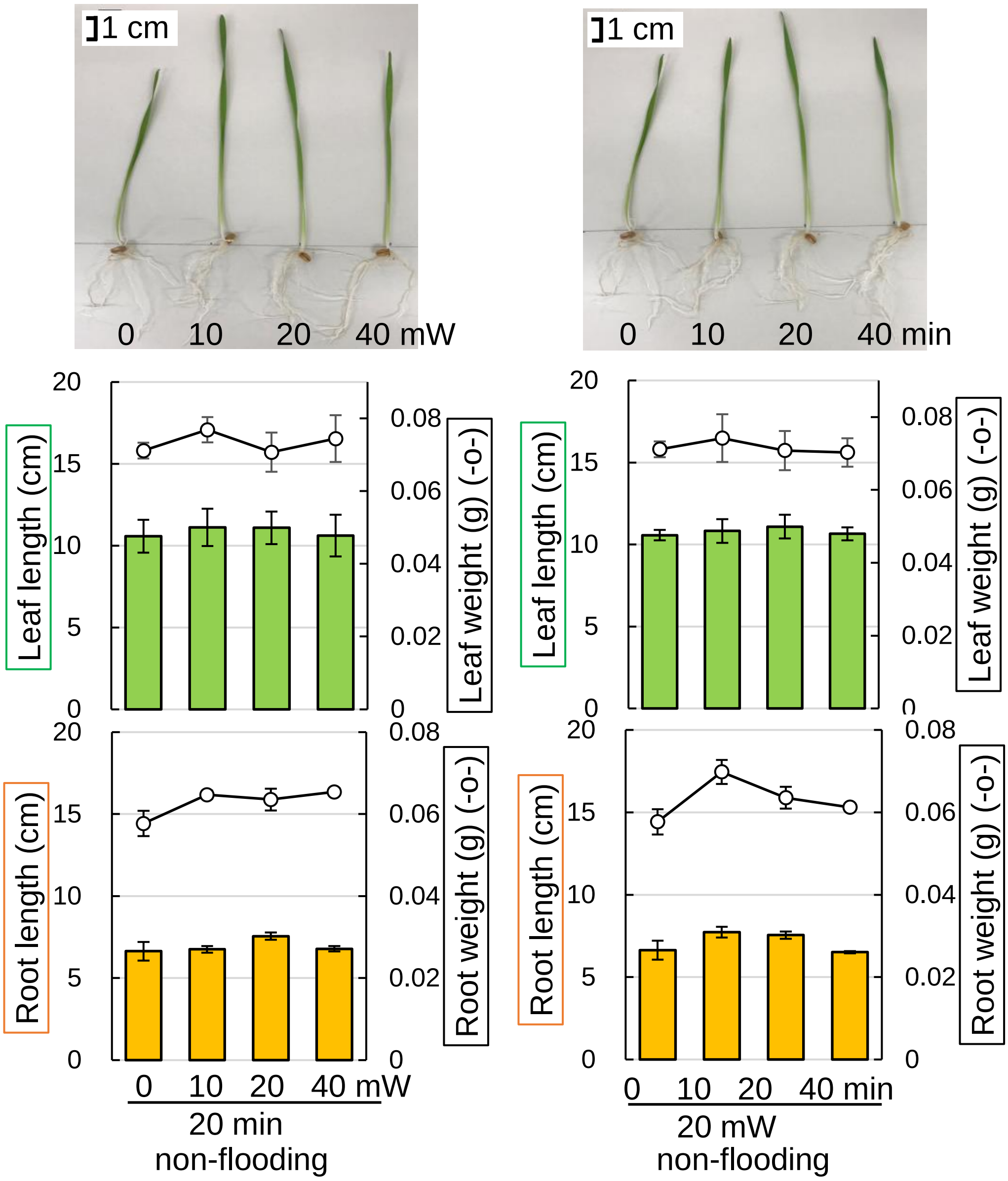

Figure S3: Morphological effect of millimeter-wave irradiation with the various power and time on wheat. Wheat seeds were irradiated with 20 mW millimeter waves for 0, 10, 20, and 40 min and sowed. For non-flooded group, samples were collected at 6 days after sowing. Leaf length (green column), leaf-fresh weight (black line graph), main-root length (orange column), and total-root weight (black line graph) were measured as morphological parameters. Bar indicates 1 cm. The data are given as the mean ± SD from 3 independent biological replicates.

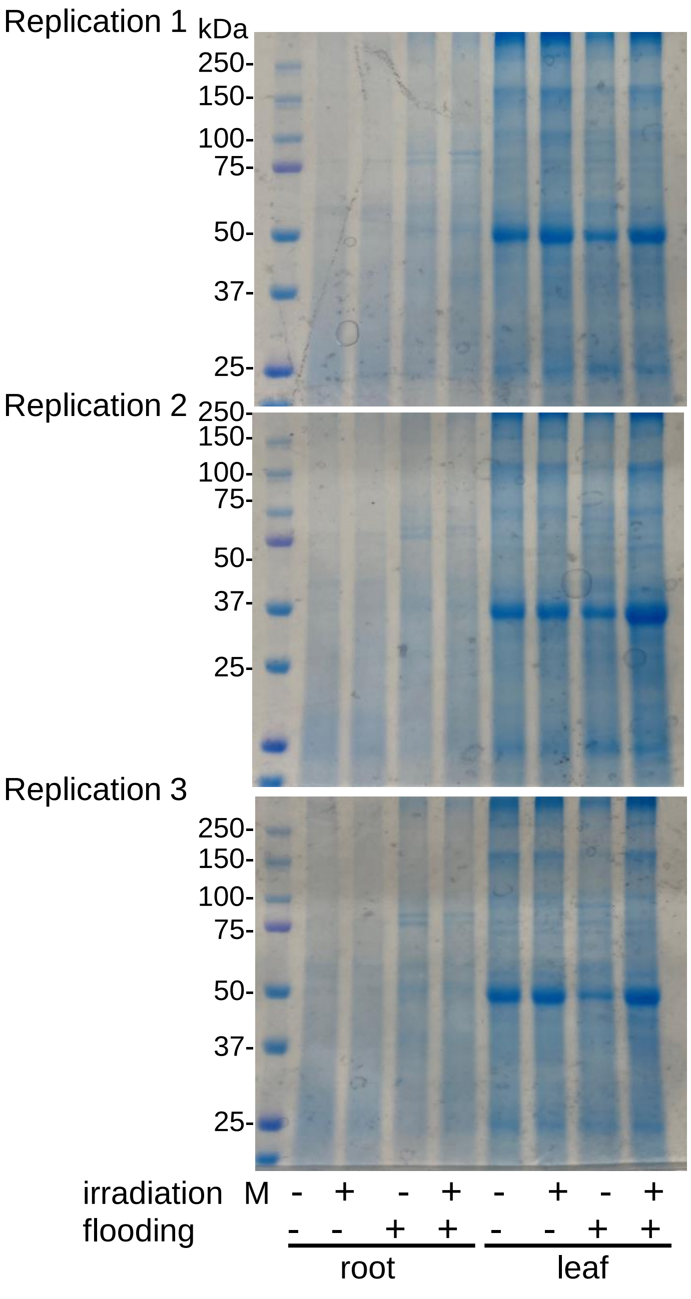

Figure S4: The gel pattern stained with the Coomassie-brilliant blue of proteins used for immunoblot analysis. Experiments were performed with biologically triplicates for each treatments. Quantified proteins (10 µg) from leaf and root were separated by electrophoresis on a 10% SDS-polyacrylamide. Coomassie brilliant blue staining was used as loading control. “M” means marker proteins.

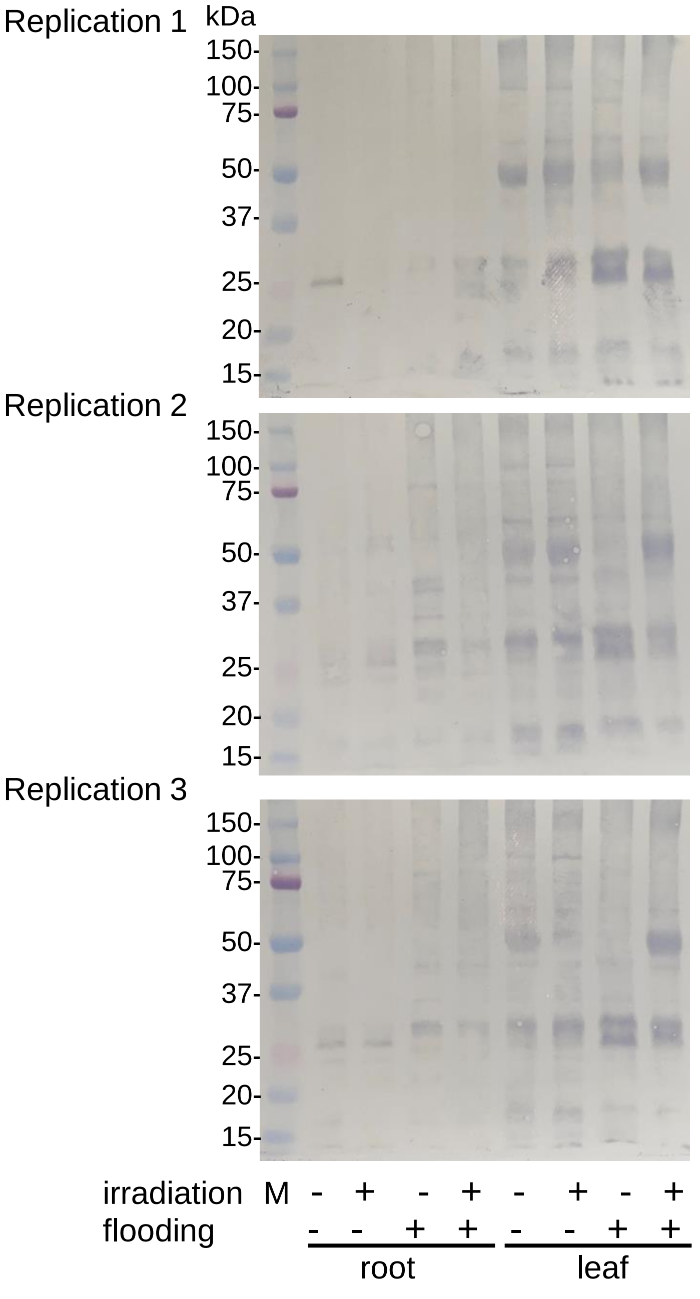

Figure S5: Immunoblot pattern of the entire membrane reacted with anti-FBA antibody used in Figure 5. “M” means marker proteins.

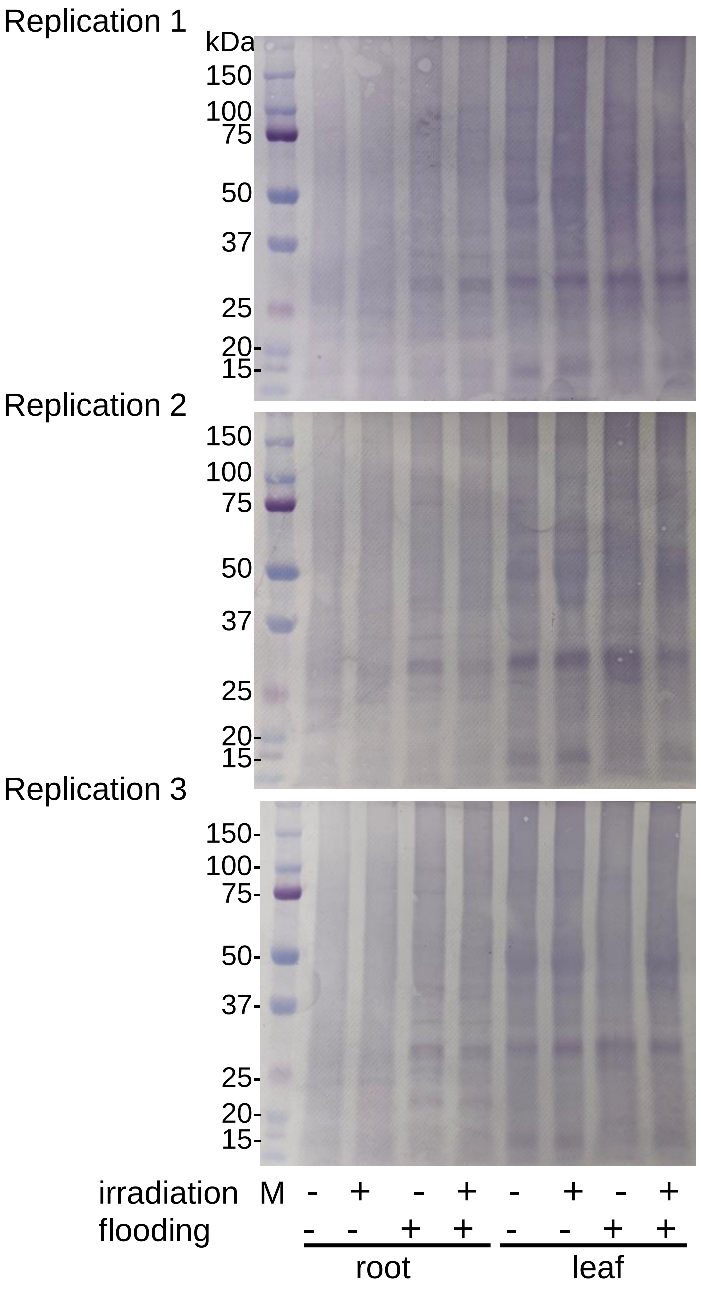

Figure S6: Immunoblot pattern of the entire membrane reacted with anti-TPI antibody used in Figure 5. “M” means marker proteins.

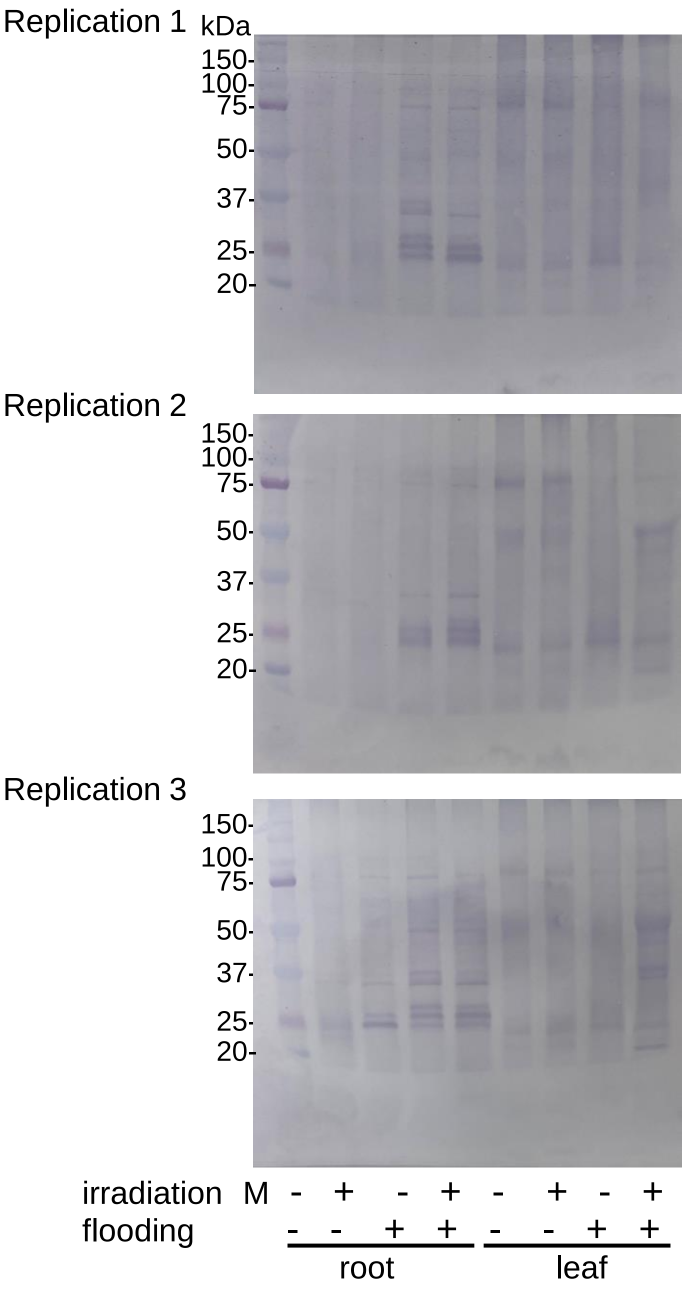

Figure S7: Immunoblot pattern of the entire membrane reacted with anti-GAPD antibody used in Figure 5. “M” means marker proteins.

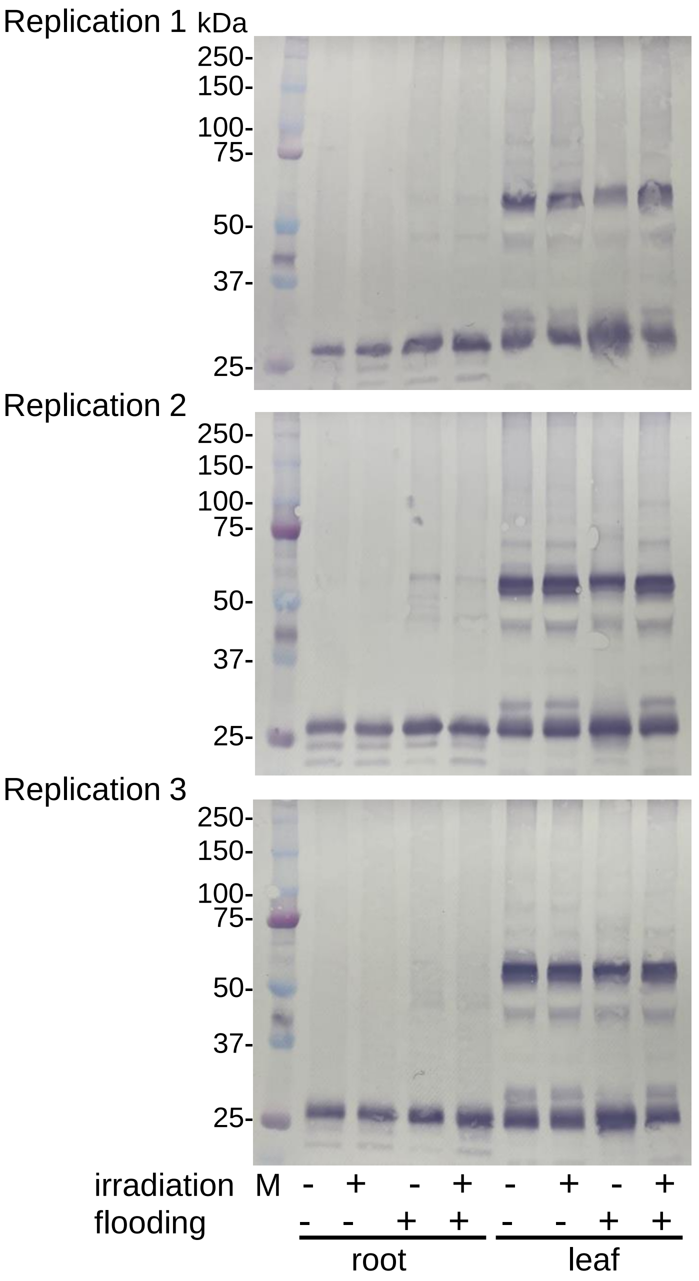

Figure S8: Immunoblot pattern of the entire membrane reacted with anti-APX antibody used in Figure 6. “M” means marker proteins.

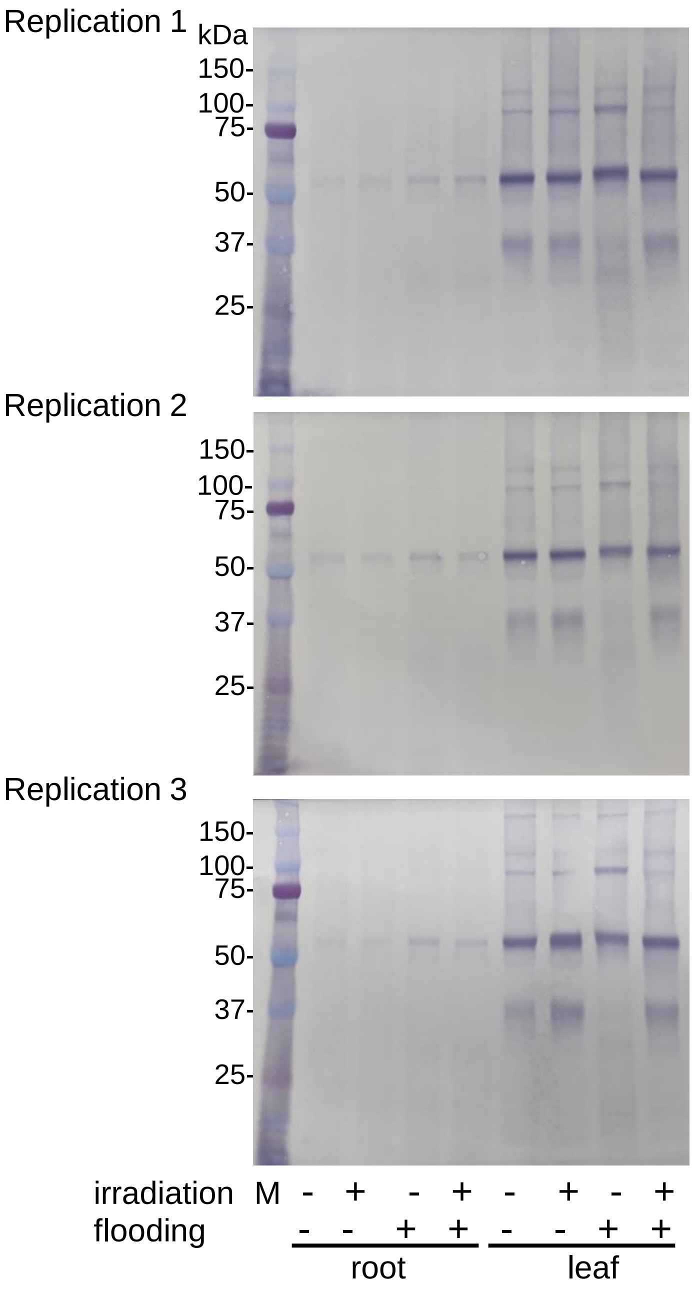

Figure S9: Immunoblot pattern of the entire membrane reacted with anti-GR antibody used in Figure 6. “M” means marker proteins.

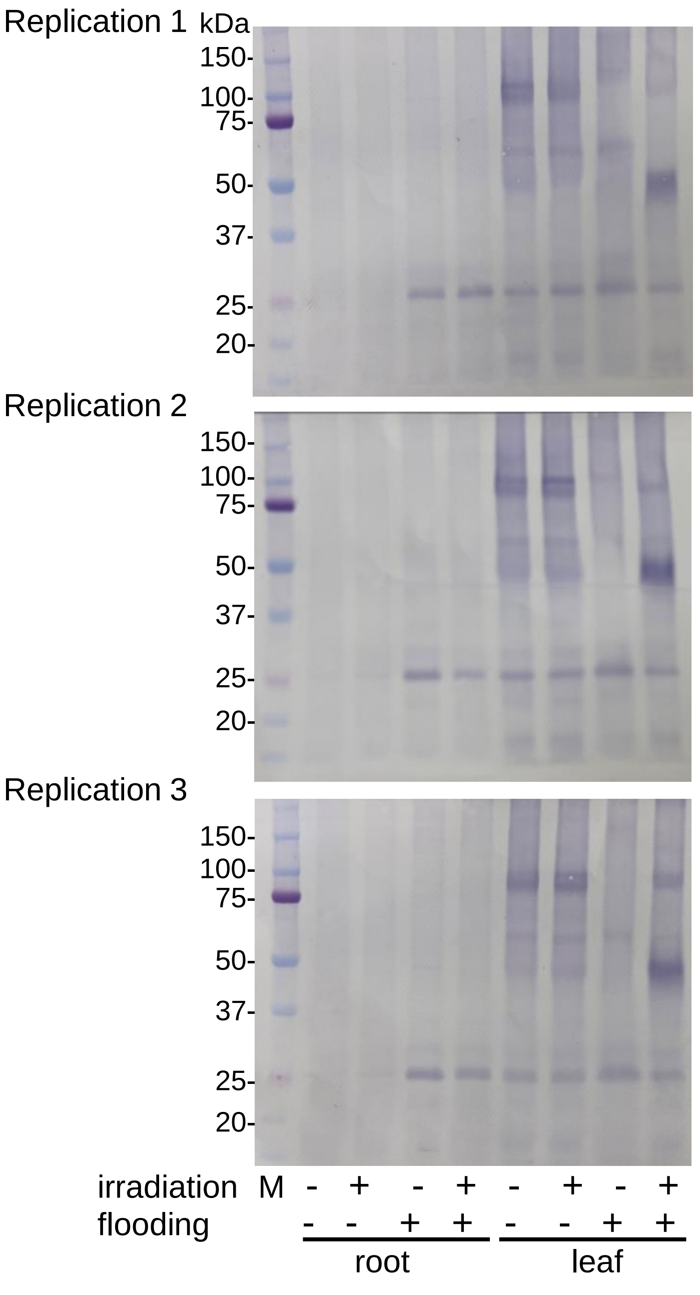

Figure S10: Immunoblot pattern of the entire membrane reacted with anti-PRX antibody used in Figure 6. “M” means marker proteins.

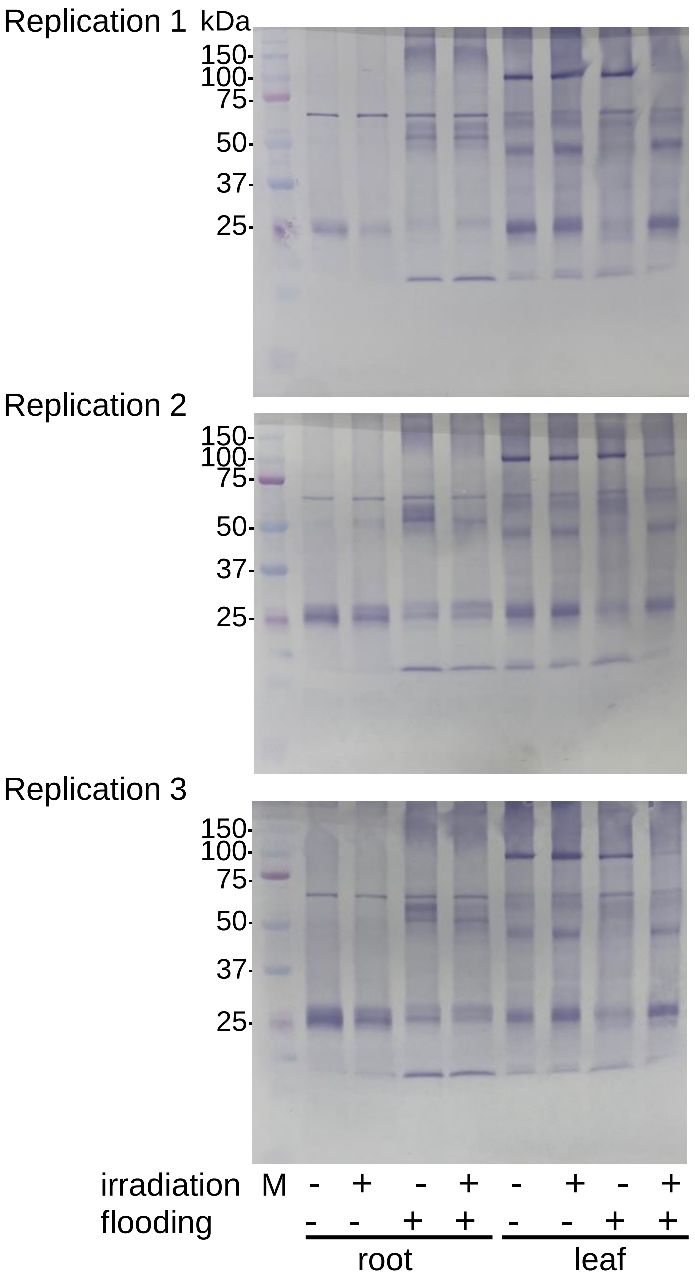

Figure S11: Immunoblot pattern of the entire membrane reacted with anti-β actin antibody used in Figure 7. “M” means marker proteins.

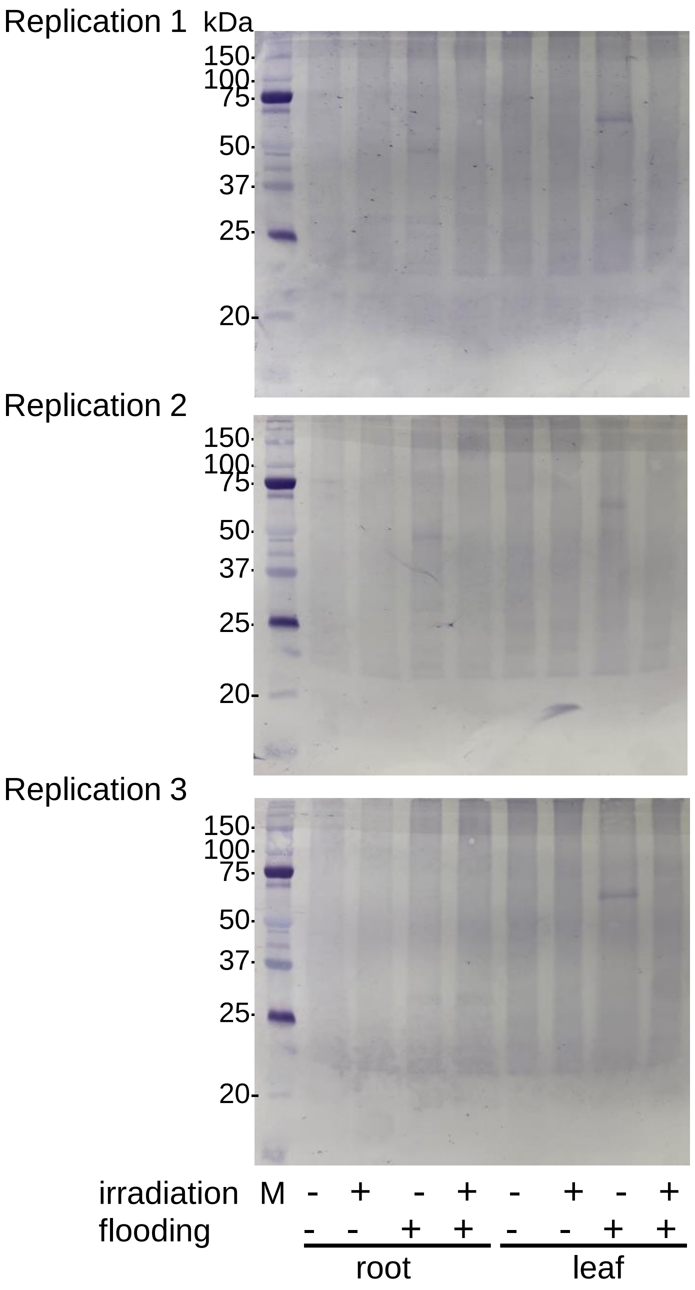

Figure S12: Immunoblot pattern of the entire membrane reacted with anti-β tubulin antibody used in Figure 7."M" means marker proteins.
