# Supplementary material for: Proteomic and Biochemical Approaches Elucidate the Role of Millimeter-Wave Irradiation in Wheat Growth under Flooding Stress
Source: Int J Mol Sci. 2022 Sep 8;23(18):10360. doi: 10.3390/ijms231810360 (PMC9499361; doi:10.3390/ijms231810360)
Supplement: Supplementary file 1 [file ijms-23-10360-s001.zip › rev Supplemental Table S1.pdf]

Table S1: Experimental procedure of quantitative proteomics used in this study.

Title	methods
Protein enrichment, reduction, alkylation, and digestion	Extracted proteins (100 µg) were adjusted to a final volume of 100 µL. The protocol of protein enrichment, reduction, alkylation, and digestion are described in the previous study (Komatsu et al., 2013). Extracted proteins (100 µg) were adjusted to a final volume of 100 µL. Methanol (400 µL) was added to each sample and mixed before addition of 100 µL of chloroform and 300 µL of water. After mixing and centrifugation at 20,000 x g for 10 min to achieve phase separation, the upper phase was discarded and 300 µL of methanol was added to the lower phase, and then centrifuged at 20,000 x g for 10 min. The pellet was collected as the soluble fraction. Proteins were resuspended in 50 mM ammonium bicarbonate, reduced with 50 mM dithiothreitol for 30 min at 56°C, alkylated with 50 mM iodoacetamide for 30 min at 37°C in the dark, and digested with trypsin and lysyl endopeptidase (Wako, Osaka, Japan) at a 1:100 enzyme/protein ratio for 16 h at 37°C. Peptides were desalted with MonoSpin C18 Column (GL Sciences, Tokyo, Japan) and acidified with 1% trifluoroacetic acid.
Protein identification using nano-liquid chromatography mass spectrometry	The LC (EASY-nLC 1000; Thermo Fisher Scientific, San Jose, CA, USA) conditions as well as the MS (Orbitrap Fusion ETD MS; Thermo Fisher Scientific) acquisition settings are described in the previous study (Li et al., 2018). The peptides were loaded onto the LC system equipped with a trap column (Acclaim PepMap 100 C18 LC column, 3 µm, 75 µm ID × 20 mm; Thermo Fisher Scientific), equilibrated with 0.1% formic acid, and eluted with a linear acetonitrile gradient (0-35%) in 0.1% formic acid at a flow rate of 300 nL min^{-1}. The eluted peptides were loaded and separated on the column (EASY-Spray C18 LC column, 3 µm, 75 µm ID x 150 mm; Thermo Fisher Scientific) with a spray voltage of 2 kV (Ion Transfer Tube temperature: 275°C). The peptide ions were detected using MS in the data-dependent acquisition mode with the installed Xcalibur software (version 4.0; Thermo Fisher Scientific). Full-scan mass spectra were acquired in the MS over 375–1500 m/z with resolution of 120,000. The most intense precursor ions were selected for collision-induced fragmentation in the linear ion trap at normalized collision energy of 35%. Dynamic exclusion was employed within 60 sec to prevent repetitive selection of peptides.
Mass spectrometry data analysis	The MS/MS searches were carried out using MASCOT (version 2.6.1, Matrix Science, London, UK) and SEQUEST HT search algorithms against the *Arabidopsis Thaliana* (UniProtKB TaxID=3702) (version 2021-02) and *Triticum aestivum* (SwissProt TaxID =4565) (version 2021-02) using Proteome Discoverer (version 2.4; Thermo Fisher Scientific). The settings of MASCOT are described in the previous study (Komatsu et al., 2021). The workflow for both algorithms included spectrum files RC, spectrum selector, MASCOT, SEQUEST HT search nodes, percolator, ptmRS, and minor feature detector nodes. Oxidation of methionine were set as a variable modification and carbamidomethylation of cysteine was set as a fixed modification. Mass tolerances in MS and MS/MS were set at 10 ppm and 0.6 Da, respectively. Trypsin was specified as protease and a maximum of two missed cleavage was allowed. Target-decoy database searches used for calculation of false discovery rate and false discovery rate was set at 1% for peptide identification.
Differential analysis of proteins using mass spectrometry data	Label-free quantification was also performed with Proteome Discoverer using precursor ions quantifiler nodes. For differential analysis of the relative abundance of peptides and proteins between samples, the free software Perseus (version 1.6.15.0) (Tyanova et al., 2016) was used. Differential analysis are described in the previous study (Komatsu et al., 2021). Proteins and peptides abundances were transferred into log2 scale. Three biological replicates of each sample were grouped and a minimum of two valid values were required in at least one group. Normalization of the abundances was performed to subtract the median of each sample. Missing values were imputed based on a normal distribution (width = 0.3, down-shift = 1.8). Significance was assessed using Student's *t*-test analysis. Protein functions were categorized using MapMan bin codes (https://mapman.gabipd.org/applications).

References:

Komatsu, S.; Han, C.; Nanjo, Y.; Altaf-Un-Nahar, M.; Wang, K.; He, D.; Yang, P. Label-free quantitative proteomic analysis of abscisic acid effect in early-stage soybean under flooding. *J Proteome Res* **2013,** *12,* 4769-4784.

Li, X.; Rehman, S.U.; Yamaguchi, H.; Hitachi, K.; Tsuchida, K.; Yamaguchi, T.; Sunohara, Y.; Matsumoto, H.; Komatsu, S. Proteomic analysis of the effect of plant-derived smoke on soybean during recovery from flooding stress. *J Proteomics* **2018,** *181,* 238–248.

Komatsu, S.; Maruyama, J.; Furuya, T.; Yin, X.; Yamaguchi, H.; Hitachi, K.; Miyashita, N.; Tsuchida, K.; Tani, M. Proteomic and biological analyses reveal the effect on growth under flooding stress of chickpea irradiated with millimeter waves. *J Proteome Res* **2021**, *20,* 4718-4727.

Tyanova, S.; Temu, T.; Sinitcyn, P.; Carlson, A.; Hein, M.Y.; Geiger, T.; Mann, M.; Cox, J. The Perseus computational platform for comprehensive analysis of omics data. *Nat Methods* **2016,** *13,* 1-740.
